# Microbial Biofilms: Structural Plasticity and Emerging Properties

**DOI:** 10.3390/microorganisms10010138

**Published:** 2022-01-10

**Authors:** Arnaud Bridier, Romain Briandet

**Affiliations:** 1 Antibiotics, Biocides, Residues and Resistance Unit, Fougères Laboratory, French Agency for Food, Environmental and Occupational Health & Safety (ANSES), 35300 Fougères, France; 2 Micalis Institute, INRAE, AgroParisTech, Université Paris-Saclay, 78350 Jouy-en-Josas, France

**Keywords:** biofilm, multicellular community, microbial functions, 3D structure, fluorescence imaging, confocal microscopy, light-sheet microscopy, single cell, multiscale approach

## Abstract

Microbial biofilms are found everywhere and can be either beneficial or detrimental, as they are involved in crucial ecological processes and in severe chronic infections. The functional properties of biofilms are closely related to their three-dimensional (3D) structure, and the ability of microorganisms to collectively and dynamically shape the community spatial organization in response to stresses in such biological edifices. A large number of works have shown a relationship between the modulation of the spatial organization and ecological interactions in biofilms in response to environmental fluctuations, as well as their emerging properties essential for nutrient cycling and bioremediation processes in natural environments. On the contrary, numerous studies have emphasized the role of structural rearrangements and matrix production in the increased tolerance of bacteria in biofilms toward antimicrobials. In these last few years, the development of innovative approaches, relying on recent technological advances in imaging, computing capacity, and other analytical tools, has led to the production of original data that have improved our understanding of this close relationship. However, it has also highlighted the need to delve deeper into the study of cell behavior in such complex communities during 3D structure development and maturation— from a single-cell to a multicellular scale— to better control or harness positive and negative impacts of biofilms. For this Special Issue, the interplay between biofilm emerging properties and their 3D spatial organization considering different models, from single bacteria to complex environmental communities, and various environments, from natural ecosystems to industrial and medical settings are addressed.

## 1. A Three-Dimensional Lifestyle

Microbial biofilms constitute a communal way of life enabling microorganisms to colonize very diverse ecological niches over the Earth—from pristine ecosystems to anthropized environments [1]. They greatly affect human well-being through their involvement in numerous beneficial ecological cycles or biotechnological applications, as well as in deleterious chronic infections or biocorrosion processes for instance. In these biological buildings, microorganisms live in close proximity embedded in extracellular polymeric substances and mostly share specific emerging properties, behaving more than simple cellular aggregates. The extracellular matrix contains a complex panel of chemically diverse molecules with various roles in biofilm development, architecture, and functions, also termed the matrixome [2]. This matrixome is highly dependent on species diversity and environmental conditions in terms of composition [3,4]. It cements cells together and highly participates in the shaping and the plasticity of biofilm spatial organization, resulting in a great diversity of biofilm architectures [5,6]. Interestingly, the term “biofilm” was recently discussed, as it evokes only one shape of the various manifestations of microbial aggregates and insufficiently reflects the complex architectural features most observed and which are at the origin of biofilm’s emerging properties [1]. The production of the extracellular polymeric matrix and the 3D expansion of biofilm indeed lead to heterogeneity of phenotypes due to chemical gradients within the structure. This heterogeneity is considered a hallmark of biofilms, along with a challenge to tackle to understand how specific functional properties emerge [7]. Nevertheless, a large number of methodologies still commonly used in biofilm studies provide only a global analysis of the whole biofilm and do not consider this spatial heterogeneity [8]. Moreover, biofilms are mostly associations of a wide range of microorganisms in natural and industrial environments, resulting in ecological diversity in addition to phenotypic heterogeneity. The ecological interactions between species also play key roles in the building of the biofilms 3D structure and functions. Structural organization between species in biofilm indeed depends on both local interactions between species-specific physiology and global environmental conditions. The multiscale analysis of biofilm organization can thus be used to better decipher the nature of ecological interactions in mixed-species communities and the underlying mechanisms [8,9,10]. In addition, some studies recently demonstrated that growth in a 3D biofilm can promote genetic diversity and may drive the evolution of mutualistic behavior, reciprocally affecting the biofilm architecture [11,12,13]. Overall, the close relationship between 3D structures and the adaptation of biofilm to internal and environmental stresses articulates the emergence of functional properties and finally drives their positive or negative impacts [14].

## 2. Advances in Biofilm 3D Characterization

In recent years, an increasing number of methodologies and approaches, supported by technological progress in imaging, modeling, and computing sciences, led to advancing further in the multiscale analysis and deciphering of the 3D structure of biofilms.

Confocal laser scanning microscopy (CLSM) is an optical microscopy technique combining a targeted laser excitation with a pinhole before detection, enabling it to collect fluorescence from a distinct focus plane. Various fluorophores can additionally be used simultaneously through multispectral acquisitions. For these reasons, CLSM was extensively used to capture both quantitative and qualitative structural data in biofilm through non-invasive 3D spatiotemporal analyses down to a single-cell scale [15,16]. The development of high-throughput screening of biofilm 3D structures, combining microtiter plates and an automatized scanning led to a dramatic increase in the number of samples studied simultaneously, thus enabling large comparison of biofilms architecture and statistical analyses [17]. Recently, light-sheet-based imaging also emerged as a successful method to characterize biofilm 3D structure. An advantage of light-sheet-based microscopy approaches is their low photobleaching, compared with CLSM, due to the weaker illumination intensity required [18]. This feature makes light-sheet microscopy (and especially dual-view light-sheet microscopy) a suitable technique to map individual cell trajectories over long durations within the biofilm and thus reveal the complex spatiotemporal dynamics of development [19]. For instance, dual-view light-sheet microscopy was elegantly used in combination with intracellular fluorescent puncta labeling and modeling to track single bacteria during *Vibrio cholerae* 3D biofilm expansion and reveal spatiotemporal patterns of development [20]. The authors highlighted the presence of two distinct cell behaviors and a collective fountain-like flow of bacteria that together govern the development of the multicellular structure. Advances in scanning electron microscopy (SEM) and the development of techniques suitable for hydrated biofilm architectural investigation such as variable pressure SEM (VP-SEM), environmental SEM (ESEM), and Ambiental SEM (ASEM), and their combination with other microscopic approaches also led to advance further in the exploration of biofilm ultrastructure and cell morphology or to compare the effect of different treatments [21,22].

Regardless of the imaging technology used, our ability to analyze biofilm structural dynamics at the single-cell scale has been greatly improved owing to the development of automated image analysis and computational tools. In a recent review, Jeckel and Drescher [23] discussed the opportunities given by recent improvements in segmentation and machine learning associated development, image cytometry, adaptive microscopy, high-dimensional data analysis, and overall image analysis methods, for the monitoring of spatiotemporal processes in microbial populations. Some image analysis tools and workflows specifically dedicated to biofilm 3D structure analysis and quantification, such as BCM3D or BiofilmQ, greatly participated in the increase in our capacities to decipher collective biofilm traits in space and time considering single-cell scale [24,25]. The development of computational methods and especially the application of artificial intelligence and machine learning to cutting-edge biofilm imaging data, and its integration with heterogeneous data such as large multi-omics datasets, for instance, provide an unprecedented opportunity to explore the link between the presence of complex structural patterns and functional properties in biofilms [26,27].

Major advances in the deciphering of biofilm development were indeed achieved owing to such integrative approaches, particularly in our ability to consider the structural heterogeneity of biofilm, which remains a major challenge to understand biofilm organization and functions. The improvement of spectral imaging techniques, in association with a highly multiplexed fluorescence in situ hybridization (FISH) approach, enabled the high-phylogenetic-resolution mapping of microbial communities [28]. The recent adaptation and application of mRNA labeling and sequential FISH (seqFISH) to bacterial populations provided access to single-cell transcriptional activities in *Pseudomonas aeruginosa* biofilms [29]. Microscale, spatial-resolved transcriptomics has indeed the potential to capture biofilm spatial and temporal heterogeneity and could further be extended to explore sub-cellular transcript organization in bacteria, and finally, delve deeper into biofilm-spatialized functional organization [30]. Moreover, tools for spatially and temporally resolved analyses of extracellular compartments of biofilms emerged. As an example, pH variation in environmental microniches in *Pseudomonas aeruginosa* and *Streptococcus mutans* biofilms was monitored in real time using pH-sensitive nanosensors. These fluorescent nanosensors revealed pH gradients during the building of 3D structures that helped to better understand the dynamic modulations of extracellular matrices during the developmental process and to potentially identify targets for biofilm control [31].

Using an innovative experimental imaging and image analysis approach based on confocal microscopy and new 3D-image segmentation techniques, Drescher et al. were able to monitor and reconstruct 3D biofilm development in the pathogen *V. cholerae* at a single-cell resolution, with up to 10,000 individuals [32]. From these observations, they better predicted developmental dynamics and 3D structure emergence of biofilm through a better deciphering of cellular interactions. Complementarily, by integrating rheological measurements, single-cell imaging by spinning disc confocal microscopy, and modeling, Zhang et al. [33] built a comprehensive view of *V. cholerae* biofilm development in confined environments using an agarose gel entrapment model. Thereby, they demonstrated that mechanical stress governs morphogenetic development and cell ordering in *V. cholerae* confined biofilms. The association of an adaptive microscopy approach with machine learning and modeling also permits the depiction of the multicellular behavior in the *Bacillus subtilis* swarming model at different scales [34]. This led to a unified, multiscale swarm expansion model, governed by cell–cell interactions, as well as by gene expression at a microscopic scale and cellular growth kinetics at the macroscopic scale.

In conclusion, the central role of 3D structural rearrangements within microbial communities in developmental strategies and functional properties underlines the need to delve deeper into our understanding of underlying cellular dynamic processes in biofilms at a multiscale level. Recent technological advances in single-cell imaging or phenotypic and genomic characterization methods, along with the development of modeling, artificial-intelligence-based, and machine learning approaches provided promising insights in this perspective, as illustrated here. Further studies and methodological development in this field should thus lead to dramatically improving our understanding of biofilm structure/function relationships in the near future.
